# Meta-analytical analysis on components released from resin-based dental materials

**DOI:** 10.1007/s00784-022-04625-4

**Published:** 2022-07-23

**Authors:** Francesco De Angelis, Nela Sarteur, Matteo Buonvivere, Mirco Vadini, Michal Šteffl, Camillo D’Arcangelo

**Affiliations:** 1 Unit of Restorative Dentistry and Endodontics, Department of Medical, Oral and Biotechnological Sciences, School of Dentistry, “G. D’Annunzio” University Chieti-Pescara, Via dei Vestini, 31, 66100 Chieti, Italy; 2 Department of Physiology and Biochemistry, Faculty of Physical Education and Sport, Charles University, Prague, Czech Republic

**Keywords:** Resin-based dental materials, Composites, Elution, Eluate, Monomer, Biocompatibility

## Abstract

**Objectives:**

Resin-based materials are applied in every branch of dentistry. Due to their tendency to release substances in the oral environment, doubts have been raised about their actual safety. This review aims to provide a comprehensive analysis of the last decade literature regarding the concentrations of elutable substances released from dental resin-based materials in different type of solvents.

**Materials and methods:**

All the literature published on dental journals between January 2010 and April 2022 was searched using international databases (PubMed, Scopus, Web of Science). Due to strict inclusion criteria, only 23 papers out of 877 were considered eligible. The concentration of eluted substances related to surface and volume of the sample was analyzed, considering data at 24 h as a reference. The total cumulative release was examined as well.

**Results:**

The most eluted substances were HEMA, TEGDMA, and BPA, while the less eluted were Bis-GMA and UDMA. Organic solvents caused significantly higher release of substances than water-based ones. A statistically significant inverse correlation between the release of molecules and their molecular mass was observed. A statistically significant positive correlation between the amount of released molecule and the specimen surface area was detected, as well as a weak positive correlation between the release and the specimen volume.

**Conclusions:**

Type of solvent, molecular mass of eluates, and specimen surface and volume affect substances release from materials.

**Clinical relevance:**

It could be advisable to rely on materials based on monomers with a reduced elution tendency for clinical procedures.

**Supplementary Information:**

The online version contains supplementary material available at 10.1007/s00784-022-04625-4.

## Introduction

The continuous demand for esthetic care and the improved properties of dental materials have led to the increasing use of resin-based composites as restorative material in dental practice. However, despite their growing popularity, many doubts have been raised over the last years about the actual safety of their use [1–5].

Resin-based composites consist of an organic matrix, reinforcing fillers, a silane coupling agent, pigments, catalysts, and inhibitors. Bis-GMA and other bisphenol A (BPA)-derived monomers are the most employed in methacrylate-based composite material fabrication, because of their relevant properties, such as flexural strength, volumetric shrinkage, water sorption/solubility, and viscosity [6]. However, they may induce genotoxicity and cytotoxicity probably through DNA damage, inhibition of cytokine release, and induction of apoptosis and necrosis [7, 8]. For example, Bis-GMA stimulates the production of PGE2 with the expression of COX2, induces pro-inflammatory activation, and increase of IL-1β, IL-6, and nitric oxide; the (co)monomer triethyleneglycoldimethacrylate (TEGDMA) is known instead to cause deoxyribonucleic acid (DNA) strand breakage [9–11].

The loss of substances from polymeric matrix mainly occurs following two mechanisms: free monomer release following the polymerization phase and intra-oral degradation [5, 12–16]. With a curing time usually not longer than 40 s and a temperature around 37 °C in the oral cavity, composites are never completely polymerized because of the propagation of the crosslinking reaction that drastically reduces the mobility of the monomers [17, 18]. Due to this incomplete polymerization, dental composites can release into the oral cavity residual monomers able to affect the biological compatibility of these materials [19–23]. As confirmed by many studies, the less the degree of conversion, the higher the amounts of elutable residual monomers [24, 25]. In addition, intra-oral degradation of resin-based restorations may induce additional release of components [26], whose majority have probably not been identified yet [27]. On one hand, they are an effect of mechanical, hydrolytic, and enzymatic scission, and, on the other hand, a result of composite aging that leads to more porosities, water sorption, and degradation [28–30]. Systemic intake of chemical substances released by resin-based restorations is possible by three main ways: diffusion to the pulp through dentinal tubules, gastro-intestinal ingestion, and uptake of volatile components in the lungs [31, 32].

In view of the progressive development of novel dental materials and the above-mentioned considerations, the idea of the present study was to review the recent literature in order to assess the quantifiable concentrations of elutable substances released from resin-based dental materials into oral environment. No up-to-date literature review regarding this argument has been published after the comprehensive analysis made by Van Landuyt et al. in 2010 [18], of which the present paper represents an update based on the literature of the last decade, aimed at taking into account also the behavior of new resin-based materials (and included elutable substances) that were not available at that time.

## Materials and methods

### Systematic review and meta-analysis protocol

The present study was conducted adhering to the guidance of PRISMA (Preferred Reporting Items of Systematic Reviews and Meta-Analyses) in order to follow a uniform and transparent methodology able to provide outcomes comparable with other meta-analytical studies.

### Research resources and strategies

The systematic research strategy was conducted by three reviewers among multiple databases: PubMed, Scopus, and Web of Science. The object of this research was all international literature with no language restrictions, published among dental journals, in the decade from January 2010 to April 2022, regarding the topic of elution of monomers from resin-based dental materials. The inserted keywords were as follows: “resin-based,” “elution,” “eluate,” “dental composite,” “HPLC,” “LC,” LC–MS,” “quantification,” “release,” “substances,” “ingredients,” “components.” The search strategy used is summarized in Table 1.

**Table 1:** Search strategy

Search strategy	
Keywords	“resin-based”, “elution”, “eluate”, “dental composite”, “HPLC”, “LC”, LC–MS”, “quantification”, “release”, “substances”, “ingredients”, “components”
Search string	(“dental composite” OR “resin-based”) AND (“elution” OR “eluate”) AND (“HPLC” OR “LC” OR “LC–MS”) AND (“quantification” OR “release”) AND (“substances” OR “ingredients” OR “components”)
Databases	PubMed, Scopus, Web of Science
Subject area	Dentistry
Language	All languages
Timeframe	January 2010–April 2022

### Data collection

All the references obtained through the above-mentioned keyword searching were collected in EndNote X9 software (Clarivate, MA, USA), where the duplicates were removed. Subsequently, all the data were loaded into Rayyan [33], an online free tool for systematic reviews. A systematic methodology was used to label all the relevant information for the exclusion or the inclusion of the individual papers. Titles and abstracts were initially screened to identify studies that potentially met the eligibility criteria. Afterwards, full texts were reviewed assessing them on the basis of the inclusion/exclusion criteria specified in the following paragraph. The decision process was performed by two independent reviewers. In case of ambiguity or disagreement between the reviewers, the final decision was reached through consultation with a third reviewer, a senior experienced researcher. The results of the selected studies were analyzed to collect mean values (and standard deviations) for the concentration of eluted molecules in the soaking solvent (both per surface and per volume of the resin sample) at a specific incubation time. Those data were then subjected to the meta-analysis.

### Inclusion and exclusion selection criteria

Only the results of the following studies were included in this research:

1. Studies investigating monomer elution in resin-based dental materials as restorative composites, bulk-fill composites, flowable composites, adhesives, resin-modified glass-ionomer cements, resin cements, CAD/CAM resin-based materials, dental sealer. Studies conducted on provisional resin-based materials, acrylic-based resins for prosthodontics and orthodontics, root canal sealers, experimental resin-based materials, and fiber-reinforced composites were not included;
2. In vitro studies. In vivo studies were excluded;
3. Studies where the results were explicitly quantified, with clear information about mean and standard deviation values. Studies with qualitative or semi-quantitative results (for example the results referred to internal standard caffeine expressed in CF%) were not included, as well as studies where standard deviation was not mentioned;
4. Studies which clearly expressed the unit of measurement of their results so as to allow an appropriate conversion into a common unit of measurement when needed. Studies using units of measurement that could not be properly converted were not included. Data for those molecules whose information about molecular weight could be gathered neither by searching the available scientific literature nor by contacting authors or material manufacturers had to be excluded as well;
5. Studies in which the incubation time for every given elution measurement was clearly specified. Only studies providing results for an incubation time of 24 h were included. If elution data after longer incubations were also provided, those results were used to calculate the mean value of total cumulative release. Results of studies where the elution after 24 h was not reported or the measurement was just performed at a shorter time were not included;
6. Studies that clearly reported the sample size (*n*);
7. Studies that clearly described the manufacturing procedure and the dimensions of the specimens, thus allowing to calculate their exact surface and volume. Studies where shape and dimensions of the specimens were unclear were not included. Studies in which the specimens were manufactured as tooth fillings for a cavity of unspecified dimensions were excluded;
8. Studies where the samples were polymerized and the methodology did not involve any pre-incubation time (for example leaving the specimen exposed to air or in any other medium) or any additional treatment (such as bleaching) before soaking;
9. Studies clearly defining the volume and quality of the solvent. Studies where that information was not provided were excluded.

In case that the paper did not provide all the required information, the full text could not be obtained or there was the need for any clarification, the corresponding authors or the manufacturer of the tested materials were contacted by email in two attempts. If it was not possible to access the necessary data in this way, then even potentially relevant studies had to be excluded.

### Recalculation

The collected elution data were inserted into MS Excel 2016 (Microsoft, WA, USA) software and prepared for statistical analysis. For those studies that did not present results numerically, but in a graph, the author of the article was contacted to supply the exact data. If it was not possible to obtain information in this way and the graph was sufficiently precise to accurately distinguish the recorded results, the online graphical tool Web Plot Digitizer [34] was used to extract them. In order to prevent data loss, if any study reported an eluate concentration “below the limit of detection,” this result was substituted by the actual value of the limit of detection specified by the author for that particular eluate. If the authors did not specify this limit, the result was substituted with the lowest measured concentration of released molecule among the results of all the included studies.

The included studies expressed the amounts of eluted monomers in several different units (mg/ml, μg/ml, ng/ml, mg/l, mmol/l, μmol/l, nmol/mm^2^). Therefore, in order to obtain uniform outcomes, it was necessary to convert them into a common unit of measurement, namely moles of eluted molecule per surface of resin specimen (μmol/mm^2^) and moles of eluted molecule per volume of resin specimen (μmol/mm^3^). The applied calculations were performed as previously suggested by Van Landuyt et al. [18] and are listed in Table 2.

**Table 2:** Calculations applied to convert all the outcomes of the included studies into common unit of measurement

	Mol/surface area [mol/m^2^]	Mol/volume [mol/m^3^]
Concentration (C) [g/l]	C [g/l] * volume solvent [l] * 1/Mm [mol/g] *1/surface area of tested specimen [1/m^2^]	C [g/l] * volume solvent [l] * 1/Mm [mol/g] *1/volume of tested specimen [1/m^3^]
Molar concentration (M) [mol/l]	M [mol/l] * volume solvent [l] * 1/surface area of tested specimen [1/m^2^]	M [mol/l] * volume solvent [l] * 1/surface area of tested specimen [1/m^3^]

### Statistical analysis

Statistical analysis was performed using IBM SPSS Statistics 24 (IBM Corp., NY, USA) statistical software. The weighted means and standard deviations (SD) of concentration (per surface and per volume of tested specimen) of each eluted substance, in the different types of incubation solvent, were calculated for the release measurements collected at 24 h and for the total cumulative release (if further measurements were performed also after 24 h). If the study provided results only at 24 h, then no total cumulative release results were calculated. In case the solvent liquid was not refreshed after every measurement, then the total cumulative release was represented by the highest measured amount of eluted substance. In case of refreshing, the total cumulative release mean value was calculated as sum of mean values of all measurements and the SD was calculated as:

$$\sqrt{\sum_{i=all\ time\ periods}(SD{i}^{2})}$$

The fixed model was used in order to calculate the weighted mean. The 95% confidence interval (*CI*) for weighted mean was also computed, with its lower and upper limits calculated as follows [35]:

$$Lower\ limit= \stackrel{-}{T.} -1.96*SE\left(\overline{T }.\right)$$

$$Upper\ limit=\stackrel{-}{T.} +1.96*SE(\overline{T }.)$$

where *T* is weighted mean for study and SE is the standard error.

Additionally, the heterogeneity was estimated using Cochran Q Statistic as:

$$Q=\sum_{i=1}^k{w_i\left(T_i-\overset-{T.}\right)}^2\mathrm{and}\;I^2=100\%\ast\frac{Q-df}Q$$

where *w*_*i*_ is the weight of each study, *T*_*i*_ is the weighted mean of each study, *Q* is the chi-squared statistic, and *df* is its degree of freedom. The interpretation of heterogeneity *I*^2^ according to Cochrane Handbook for Systematic Reviews is as follows: 0 to 40%, might not be important; 30 to 60%, may represent moderate heterogeneity; 50 to 90%, may represent substantial heterogeneity; and 75 to 100%, considerable heterogeneity [36].

The difference between weighted means of six most frequently detected monomers (Bis-EMA, Bis-GMA, BPA, HEMA, TEGDMA, UDMA) in water-based and organic solvents was examined through *z*-test. Pearson’s correlation coefficient was applied in order to assess correlations between the release of these six monomers and molecular mass, surface of specimen, volume of specimen, and volume of incubation solvent. The correlation coefficients were interpreted according to the following scale [37]: 0.00–0.10 as negligible correlation, 0.10–0.39 as weak, 0.40–0.69 as moderate, 0.70–0.89 as strong, 0.90–1.00 as very strong correlation.

## Results

### Systematic review

**Fig. 1:**
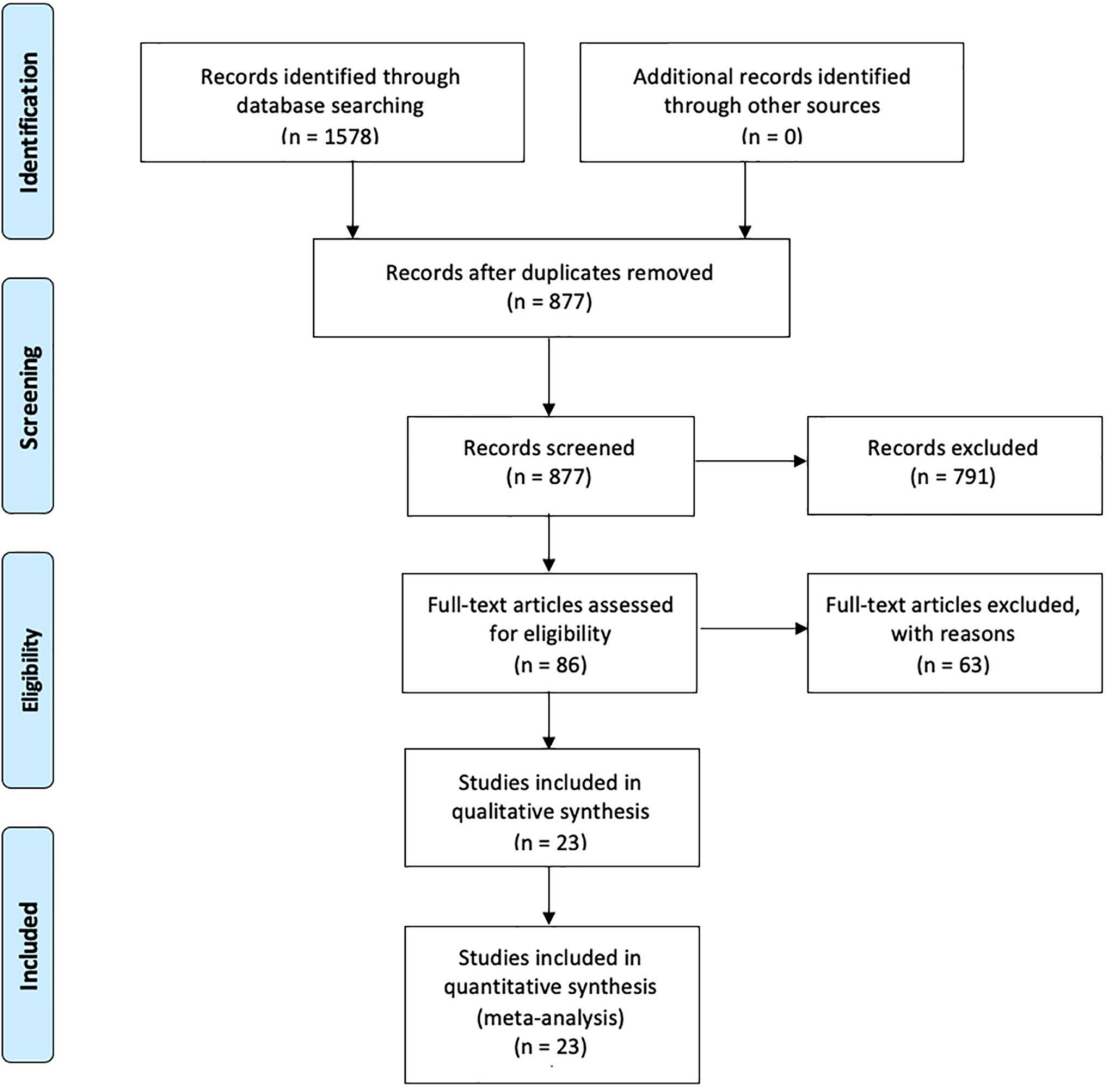
PRISMA flow diagram used for record retrieval and inclusion

The electronic research through three different databases (PubMed, Scopus, Web of Science), inserting the keywords separately or in combination, generated a total of 1578 references, which were reduced to 877 after the duplicate removal. After the examination of titles and abstracts, 791 studies were excluded because of their study design incompatibility with this review (*n* = 653), wrong publication type (*n* = 70) or wrong type of material undergoing the research (*n* = 68). A total of 86 potentially relevant studies accessed the full-text evaluation phase resulting in a final number of 63 articles excluded with reasons [3, 38–87] (Table 3) and 23 publications included for further quantitative assessment (Fig. 1). A list of the included studies and their basic information is shown in Table 4. Extended information about the same studies, including more details regarding the protocols and the methodologies used, is provided in Online Resource 1.

**Table 3:** List of references excluded after full text evaluation because they did not match some specific inclusion criteria

Excluded references	Reasons for exclusion
Bandarra 2020, Kopperud 2011, Phan 2014, Randolph 2014, Wolff 2016	Temporary provisional resin-based materials, acrylics for prosthodontics and orthodontics, root canal sealers, experimental resin-based materials, fibre-reinforced composites
Manojlovic 2011, Gul 2021	In vivo studies, reviews, and meta-analysis
Grenade 2017, Bationo 2016, Durner 2015	Studies with qualitative analysis
Durner 2010, Durner 2012, Kolaouzidou 2018, Janani 2021, Roussou 2021	Studies with semi-quantitative analysis
Meyer Lueckel 2020	Results of Mean and Standard Deviation not specified
Dursun 2016	Unit of analysis not clearly specified or unconvertible to common unit for this study
Pelourde 2018, Pongprueksa 2014, Cokic 2018, Durner 2014, Hussain 2017, Illie 2014, Kerezoudi 2016, Kerezoudi 2019, Putzeys 2019, Putzeys 2020, Randolph 2014, Yang 2018, Rothmund 2015, Alamoush 2021, De Nys 2021, Kincses 2021, Shahabi 2021, Aldhafyan 2022	Studies where the analysis at 24 h was not performed
Durner 2014, Durner 2015, Durner 2012, Illie 2014, Tuna 2010	Volume of solvent not specified
Wolff 2016, Hatipoglu 2019, Durner 2020, Durner 2011, Polydorou 2013, Purushothaman 2015, Sunitha 2011, Wolff 2016, Manojlovic 2011, Tak 2015, Durner 2021, Jo 2021	Studies where on the basis of the provided information about specimens shape and dimensions, it was not possible to calculate the surface and volume exposed to solvent for elution
Deviot 2018, Khalid 2018, Song 2020, Bartucigil 2020, Grenade 2017, Hope 2016, Gul 2019, Schuster 2015, Schuster 2016, Durner 2011, Ylmaz 2022	Studies involving pre-incubation time or pre-treatment before the analysis
Polydorou 2011, Mavishna 2020, Tabatabaei 2011	No full text accessible

**Table 4:** Included studies

	Authors	Title	Journal	Resin Based Material	Analytical method	Units	Quantified eluates	Incubation solvent
1	*Alshali RZ, Salim NA, Sung R, Satterthwaite JD, Silikas N*	Analysis of long-term monomer elution from bulk-fill and conventional resin-composites using high performance liquid chromatography	Dent Mater 2015;31:1587–1598	Resin-based filling materials (bulk fill and conventional filling materials)	HPLC	μg/ml	BisEMA BisGMA DEGDMA HDDMA SDR-UDMA TCD-DI-HEA TEGDMA UDMA	70% ethanol/water solution Distilled water Artificial saliva Volume: 1.5 ml Temperature: 37 °C Analysis intervals: 24 h, 1 month, 3 months
2	*Atabek D, Aydintug I, Alaçam A, Berkkan A*	The Effect of Temperature on Bisphenol: An Elution from Dental Resins	J Contemp Dent Pract 2014;15(5):576–80	Resin-based composites (filling materials containing and non-containing BPA)	HPLC	μg/ml	BPA	75% ethanol/water solution Volume: 2 ml Temperature: 37 °C Analysis intervals: 1, 6, 24 h and 2, 3, 4, 5, 6 days
3	*Barutcigil K, Dündar A, Batmaz SG, Yıldırım K, Barutçugil Ç*	Do resin based composite CAD/CAM blocks release monomers?	Clin Oral Investig 2021;25(1):329–336	Resin-based CAD/CAM materials	HPLC	μg/ml	BisEMA BisGMA HEMA TEGDMA UDMA	75% ethanol/water solution Volume: 5 ml Temperature: room temperature Analysis intervals: 1 h, 24 h, and 90 days
4	*Bationo R, Rouamba A, Diarra A, Beugré-Kouassi MLA, Beugré JB, Jordana F*	Cytotoxicity evaluation of dental and orthodontic light-cured composite resins	Clin Exp Dent Res 2021;7(1):40–48	Resin-based composites (orthodontic and filling materials)	GC/MS	μg/ml	BPA	Fibroblastic medium Volume: 400 μl Temperature: 37 °C Analysis interval: 24 h
5	*BezginT., CimenC., Ozalp N*	Evaluation of Residual Monomers Eluted from Pediatric DentalRestorative Materials	Biomed Res Int. 2021 Sep 16;2021:6,316,171	Resin-based filling materials (bulk fill, conventonal filling materials, compomer)	HPLC	μg/ml	BisGMA HEMA TEGDMA UDMA	75% ethanol/water solution Volume: 1.5 ml Temperature: room temperature Analysis intervals: 24 h, 48 h, 72 h
6	*Cebe MA, Cebe F, Cengiz MF, Cetin AR, Arpag OF, Ozturk B*	Elution of monomer from different bulk fill dental composite resins	Dental Materials 2015;31(7):e141-9	Resin-based composites (bulk fill)	HPLC	μmol/l	BisEMA BisGMA HEMA TEGDMA	75% ethanol/water Volume: 0.5 ml Temperature: room temperature Analysis intervals: 10 min, 1 h, 24 h, 30 days
7	*De Nys S, Putzeys E, Vervliet P, Covaci A, Boonen I, Elskens M, Vanoirbeek J, Godderis L, Van Meerbeek B, Van Landuyt KL, Duca RC*	A novel high-sensitivity UPLC-MS/MS method for the evaluation of Bisphenol A leaching from dental materials	Scientific Reports 2018;8(1):6981	Resin-based composites (filling materials)	UPLC—MS/MS	pmol/mm^2^	BPA	Artificial saliva Volume: 1 ml Temperature: 37 °C Analysis intervals:1 to 7 days
8	*Durner J, Stojanovic M, Urcan E, Spahl W, Haertel U, Hickel R, Reichl FX*	Effect of hydrogen peroxide on the three-dimensional polymer network composites	Dent Mater 2011;27(6):573–80	Resin-based composites (filling materials)	GC/MS	μmol/l	BisEMA BPA DDDMA EGDMA MAA TEGDMA 2PEA BHT BL CSA DCHP DEDHTP DMABEE HQME Tinuvin P TPP TPSb	Methanol Volume: 100 mg/ml Temperature: 37 °C Analysis intervals:1 day, 7 days
9	*Furche S, Hickel R, Reichl FX, van Landuyt K, Shehata M, Durner J*	Quantification of elutable substances from methacrylate based sealers and their cytotoxicity effect on with human gingival fibroblasts	Dent Mater 2013;29(6):618–25	Resin-based dental sealers	GC/MS	μmol/l	TEGDMA BHT CQ Tinuvin P TPSb	Distilled water Methanol Temperature: 37 °C Volume: 100 mg/ml Analysis intervals:1 d, 3 d, 7 d
10	*Gul P, Alp HH, Özcan M*	Monomer release from bulk-fill composite resins in different curing protocols	J Oral Sci 2020;62(3):288–292	Resin-based composites (bulk fill)	HPLC	μmol/l	BisGMA BPA HEMA TEGDMA UDMA	75% ethanol/water solution Volume: 1 ml Temperature: 4 °C Analysis interval: 24 h
11	*Hatipoglu, O.; Turumtay, E. A.; Saygin, A. G*	EEvaluation of monomer elution, microhardness, and roughness of experimental dental composite resins prepared from Bis-EFMA, a novel monomer system	Polymer Composites—Volume 43, Issue 1, pp. 584–592	Resin-based composites (experimental and commercial filling materials)	HPLC–DAD	μmol/l	BisEFMA BisGMA TEGDMA UDMA	75% ethanol/water solution Volume: 1.5 ml Temperature: 37 °C Analysis intervals: 1, 3, 7 days
12	*Högg C, Maier M, Dettinger-Maier K, He X, Rothmund L, Kehe K, Hickel R, Reichl FX*	Effect of various light curing times on the elution of composite components	Clin Oral Investig 2016;20(8):2113–2121	Resin-based composites (filling materials)	GC/MS	μmol/l	DEGDMA EGDMA TEGDMA BHT CQ HMBP	Methanol Distilled water Volume: 1 ml Temperature: 37 °C Analysis interval: 1, 3, 7 days
13	*Kurt A, Altintas SH, Kiziltas MV, Tekkeli SE, Guler EM, Kocyigit A, Usumez A*	Evaluation of residual monomer release and toxicity of self-adhesive resin cements	Dent Mater J 2018;37(1):40–48	Resin-based self-adhesive cements	HPLC	μmol/l	TEGDMA UDMA	Artificial saliva Volume: 1.5 ml Temperature: 37 °C Analysis intervals: 1 h, 24 h, 72 h
14	*Kwon HJ, Oh YJ, Jang JH, Park JE, Hwang KS, Park YD*	The effect of polymerization conditions on the amounts of unreacted monomer and bisphenol A in dental composite resins	Dent Mater J 2015;34(3):327–35	Resin-based composites (filling materials)	HPLC	mg/l	BisGMA BPA TEGDMA UDMA	Methanol Volume: 1 ml Temperature: room temperature Analysis interval: 24 h
15	*Mourouzis P, Andreasidou E, Samanidou V, Tolidis K*	Short-term and long-term release of monomers from newly developed resin-modified ceramics and composite resin CAD-CAM blocks	J Prosthet Dent 2020;123(2):339–348	Resin-based CAD CAM blocks	HPLC	ng/μl	BPA UDMA TEGDMA DMA BisEMA BisGMA	Distilled water Ethanol solution Volume: 8 ml Analysis intervals: 1 d, 7 d, 30 d, 60 d
16	*Nocca G, Iori A, Rossini C, Martorana GE, Ciasca G, Arcovito A, Cordaro M, Lupi A, Marigo L*	Effects of barriers on chemical and biological properties of two dual resin cements	Eur J Oral Sci 2015;123(3):208–14	Resin-based dual cure cements	HPLC	nmol/l	TEGDMA	DMEM (Dulbecco’s modified Eagle’s medium) Volume: 2 ml Temperature: 37 °C Analysis interval: 24 h
17	*Reichl FX, Löhle J, Seiss M, Furche S, Shehata MM, Hickel R, Müller M, Dränert M, Durner J*	Elution of TEGDMA and HEMA from polymerized resin-based bonding systems	Dent Mater 2012;28(11):1120–5	Adhesive systems	GC–MS	μg/ml	TEGDMA HEMA	Distilled water Methanol Volume: 1 ml Temperature: 37 °C Analysis intervals: 1, 2, 5, 20, 30 d
18	*Rothmund L, Reichl FX, Hickel R, Styllou P, Styllou M, Kehe K, Yang Y, Högg C*	Effect of layer thickness on the elution of bulk-fill composite components	Dent Mater 2017;33(1):54–62	Resin-based composites (bulk fill)	GC/MS	μg/ml	BisEMA HEMA TEGDMA DDDMA HPMA BEMA BHT CQ CSA DDHTP DMABEE HMBP	Distilled water Methanol Volume: 1 ml Temperature: 37 °C Analysis intervals: 24 h, 7 days
19	*Schuster L, Rothmund L, He X, Van Landuyt KL, Schweikl H, Hellwig E, Carell T, Hickel R, Reichl FX, Högg C*	Effect of opalescence (R) bleaching gels on the elution of dental composite components	Dent Mater 2015;31(6):745–57	Resin-based composites (filling materials)	GC/MS	μmol/l	BPA EGDMA HEMA UDMA DDDMA DODDMA benzaldehyd BHT BPE DCHP| DDHTP DMABEE HMBP Tinuvin P	Distilled water Methanol Volume: 1 ml Temperature: 37 °C Analysis intervals: 1 day, 7 days
20	*Schuster L, Reichl FX, Rothmund L, He X, Yang Y, Van Landuyt KL, Kehe K, Polydorou O, Hickel R, Högg C*	Effect of Opalescence (R) bleaching gels on the elution of bulk-fill composite components	Dent Mater 2016;32(2):127–35	Resin-based composites (bulk fill)	GC/MS	μmol/l	HEMA BHT DCHP DMABEE Tinuvin P TMPTMA	Distilled water Methanol Volume: 1 ml Analysis intervals: 1 day, 7 days
21	*Susila AV, Balasubramanian V*	Correlation of elution and sensitivity of cell lines to dental composites	Dent Mater 2016;32(3):e63-72	Resin-based composites (filling materials)	LC–MS	μg/ml	Bis EMA Bis GMA TEGDMA UDMA BPA CQ Ref Monomer PI	Distilled water Ethanol/water solution MEM (minimum essential medium) Volume: 1 ml Temperature: 37 °C Analysis intervals: 1 day, 2 months
22	*Tichý A., Šimková M., VrbováR., Roubíčková A., Dušková M., Bradna M*	Bisphenol A release from dental composites and resin-modified glass ionomers under two polymerization conditions	Polymers 2022;14,46	Resin-based filling materials and resin-modified glassionomers	LC–MS	ng/g	BPA	Methanol Artificial saliva Volume: 2 ml Temperature: 37 °C Analysis intervals: 1, 4, 9, 16, 35, 65, 130, 260 days
23	*Yang Y, Reichl FX, Ilie N, Shi J, Dhein J, Hickel R, Högg C*	Antioxidants as novel dental resin-composite component: Effect on elution and degree of conversion	Dent Mater 2019;35(4):650–661	Resin-based composites (filling materials)	GC/MS, HPLC/UV/DAD, HPLC/FLD, FTIR	μmol/l	HEMA TEGDMA BHT CQ CSA DDHTP DMABEE HMBP Tinuvin P	Distilled water Methanol Volume: 1 ml Temperature: 37 °C Analysis intervals: 1 day, 7 day

The studied resin-based materials were all commercial products commonly used in dental treatments, most often resin-based composites for restorations, but also adhesives, resin modified glass-ionomer cements, resin cements, CAD/CAM resin-based materials, and dental sealers. The authors always indicated the product designation and manufacturer.

Most of the studies included in the present research had a similar protocol. When dealing with materials that had to undergo a polymerization process, authors applied them inside prefabricated molds with specified forms and dimensions. These were predominantly discs of various diameter and thickness (in most cases 5 mm diameter and 2 mm thickness). Subsequently, molds top were covered with glass slides or matrix strips. The polymerization was performed with different types of polymerization lamps (halogen and led lamps) operated with various intensity and power settings. However, a LED lamp on standard curing mode (with an output irradiance of 1200 mW/cm^2^ and a wavelength range of 430–480 nm) was prevalent through the included studies. The quality of irradiance was often confirmed with calibrated radiometer systems. The polymerization time respected the manufacturer’s recommendations and the distance was reduced to a minimum (mostly 0 mm). The protocol of some studies also included a surface polishing phase [80, 88, 89].

Once all specimens were prepared, they were soaked in several types of solvents divided into water-based media (distilled water, artificial saliva and cell culture mediums as fibroblast grow medium, minimum essential medium, and Dulbecco’s Modified Eagle Medium) and organic media (methanol, ethanol, and their dilutions with water). The most used solvents were distilled water, 75% ethanol-distilled water solution, and methanol. The volume of solvent used among the studies ranged between 0.4 and 10 ml (most commonly 1 ml). Many authors performed the elution measurements of multiple types of materials in multiple solvents simultaneously.

In some studies, multiple measurements were performed at different soaking times refreshing the solvent after each measurement; their results showed, therefore, a decreasing tendency over time. In other studies, the solvent was not refreshed: these papers reported increasing results due to the aggregate amounts of leached molecules over time. In all the selected studies, a common measurement time interval of 24 h was observed: even though some studies investigated the elution of monomers and additives after shorter times, such as after 1 h [53, 90], and other works followed the results up to 1 [53, 90, 91], 2 [92, 93], or 3 months [80, 94], the majority observed the elution between 1 day and 1 week.

Although some authors used gas chromatography/mass spectrometry (GC/MS) [46, 62, 72, 83, 84, 89, 91, 95–99] or liquid chromatography/mass spectrometry LC/MS [92, 100, 101], the release of monomers and additives was prevalently detected by the method of high performance liquid chromatography (HPLC). A list of the included studies together with detailed information on the technical parameters of their protocols is given in Online Resource 1. All the molecules detected in the solvents, mainly monomers and additives (as initiators, inhibitors, etc.), are listed in Table 5. The most detected molecules were as follows: triethylene glycol methacrylate (TEGDMA), bisphenol A diglycidyl methacrylate (Bis-GMA), ethoxylated bisphenol A glycol dimethacrylate (Bis-EMA), 2-hydroxyethyl methacrylate (HEMA), urethane dimetacrylate (UDMA), bisphenol A (BPA).

**Table 5:** Molecules detected in solvents

Abbreviation name	Full name	Function	Molecular mass (g.mol^−1^)	Number of studies that detected the molecule	Number of records
2PEA	2-phenoxyethylacrylate	Other additive substances	192.21	1/23	1
BEMA	Behenyl Methacrylate	Ester of methacrylic acid	390.45	1/23	3
BHT	Butylhydoxytoluen	Free-radical polymerization inhibitor	220.36	6/23	31
Bis- EMA	Ethoxylated bisphenol Aglycol dimethacrylate	Monomer	452	7/23	65
Bis-EFMA	9,9-Bis[4-(2-hydroxyethoxy)phenyl]fluorene)	Monomer	546.6	1/23	20
Bis-GMA	Bisphenol A diglycidyl methacrylate	Monomer	512.59	9/23	120
BL	Benzil	Other additive substances	210	1/23	2
BPA	Bisphenol A	Contaminant	228.29	11/23	81
BPE	Phenylbenzoat	Other additive substances	198.22	1/23	1
CQ	Camphorquinone	Photoinitiator	166	5/23	39
CSA	Campheracid anhydride	Other additive substances	182	2/23	18
DCHP	Dicyclohexyl phthalate	Softener	330	3/23	4
DDDMA	1,10-decanediol Dimethacrylate	Monomer	310	2/23	5
DDHTP	Diethyl 2,5-dihydroxytrepthalate	Other additive substances	254.24	2/23	8
DEDHTP	Diethyl 2,5-dihydoxyterepthalate	Other additive substances	254.08	1/23	2
DEGDMA	Di(ethylene glycol) dimethacrylate	Monomer	242	1/23	2
DMA	N-dimethylacrylamide	Polymer syntesis intermediate	99.13	1/23	10
DMABEE	4-N,N-Dimethylaminobenzoic acid ethyl ester	Coinitiator	193	3/23	20
DODDMA	1,12-Dodecanediol dimethacrylate	Comonomer	338.5	1/23	1
EGDMA	Ethylenglycol dimethacrylate	Monomer	198	3/23	7
HDMMA	1,6-Hexandiol dimethacrylate	Comonomer	254.32	1/23	2
HEMA	2-hydroxyethyl methacrylate	Monomer	130,14	9/23	64
HMBP	2-Hydroxy-4-methoxy benzophenone	Photostabilizer	228	4/23	20
HPMA	2/3-Hydroxypropyl methacrylate	Comonomer	144.17	1/23	6
HQME	Hydroquinone methyl ether	Other additive substances	124.13	1/23	3
MAA	Methacrylic acid	Monomer degradation product	100.12	1/23	3
MMA	Methylmethacrylate	Monomer	86	1/23	3
PI	P-(octoyloxy phenyl) phenyl iodonium hexafluoro antimonate	Other additive substances	641.5	1/23	1
SDR UDMA	Sure fill flow (SDR) modified UDMA	Modified monomer	849	1/23	1
TCD-DI-HEA	Bis (acryloyloxymethyl) tricyclo [5.2.1.02,6] decane	Other additive substances	304.38	1/23	2
TEGDMA	Triethylene glycol dimethacrylate	Monomer	286.32	17/23	140
TIN P	Tinuvin P	Photostabilizer	225	3/23	7
TMPTMA	Trimethylpropane trimethacrylate	Comonomer	338.4	1/23	2
TPP	Triphenyl phosphane	Other additive substance—Impurity	262	1/23	1
TPSb	Triphenyl stibane	Catalysator residual of Bis-GMA synthesis	352	1/23	6
UDMA	Diurethane dimethacrylate	Monomer	470.56	10/23	130

In some cases [80, 93–95, 99–102], the authors reported an elution that was below the limit of detection: to quantify such an information, the actual limit of detection value reported in the study was used as raw datum. In case the actual limit of detection was not published [72, 90, 95, 96, 99, 102–104] and not retrievable from the authors, it was substituted by the lowest measured result of concentration for every specific molecule among the included studies.

The present review included also studies comparing substance elution under various conditions, such as different solvent temperatures [88], different polymerization times, distances and settings [96, 101, 104, 105], polymerization through a barrier [106], experimental addition of antioxidants into the investigated material [98], and application of bleaching agents [83, 84, 103]. For those studies, however, only the data coming from the control groups, including specimens manufactured in basic conditions and strictly following the producer’s instructions, were collected.

### Release

Tables 6 and 7 summarize the achieved results as weighted means (and SD) for the release measurements collected at 24 h and for the total cumulative release, per surface and per volume of tested specimens.

**Table 6:** Weighted means (and SD) for the release measurements collected at 24 h and for the total cumulative release per surface of tested specimens (μmol/mm^2^)

Eluate	24 h release								Total cumulative release							
	Number of studies	Number of records	% > DL	Weighted mean	SD	Lower *CI*	Upper *CI*	*I*^2^	Number of studies	Number of records	% > DL	Weighted mean	SD	Lower	Upper	*I*^2^
Water-based incubation solution																
BHT	4	17	94.1	0.0172	0.0071	0.0092	0.0252	86.1	4	17	94.1	0.0182	0.0079	0.0093	0.0271	87.3
BisEMA	4	27	29.2	0.0545	0.0029	0.0520	0.1065	98.0	4	24	29.2	0.0686	0.0029	0.0661	0.1347	98.5
BisGMA	3	21	9.5	0.0122	0.0125	0.0013	0.0135	85.7	3	21	9.5	0.0281	0.0094	0.0199	0.0480	98.3
BPA	2	8	37.5	0.0105	0.0075	0.0039	0.0144	84.3	2	8	37.5	0.2147	0.0947	0.1075	0.3219	81.0
CQ	4	21	100	0.0339	0.0051	0.0281	0.0396	3.5	4	21	100	0.0398	0.0056	0.0335	0.0461	73.2
CSA	2	9	100	0.0312	0.0036	0.0271	0.0352	95.4	2	9	100	0.0342	0.0043	0.0293	0.0391	97.2
DEGDMA	1	2	50.0	4.1902	0.7575	-	-	-	1	2	50.0	7.1854	0.8643	-	-	-
DMA	1	5	20.0	0.0099	0.0006	-	-	-	1	5	20.0	0.0395	0.0006	-	-	-
DMABEE	3	10	100	0.1195	0.0436	0.0701	0.1689	0	3	9	100	0.1405	0.0393	0.0961	0.1850	10.2
HDMMA	1	1	100	0.7823	-	-	-	-	1	1	100	2.3468	-	-	-	-
HEMA	2	6	100	0.2842	0.1878	0.2716	0.2967	97.2	2	6	100	0.6475	0.0113	0.6347	0.6603	98.3
HMBP	1	9	100	0.1645	0.0131	-	-	-	1	9	100	0.1781	0.0162	-	-	-
HPMA	1	3	100	1.2645	0.1827	-	-	-	1	3	100	1.0406	0.6136	-	-	-
PI	1	1	100	0.0273	-	-	-	-	1	1	100	0.0419	-	-	-	-
SDR UDMA	1	1	100	0.2343	-	-	-	-	1	1	100	0.7030	-	-	-	-
TCD-DI-HEA	1	1	100	0.1205	-	-	-	-	1	1	100	0.3358	-	-	-	-
TEGDMA	5	14	92.9	0.0339	0.0310	0*	0.0690	65.2	4	28	78.6	0.0463	0.0353	0.0063	0.0862	95.3
TMPTMA	1	1	100	0.0401	-	-	-	-	1	1	100	0.0616	-	-	-	-
TPSb	1	6	67.0	0.0096	0.0063	-	-	-	1	6	67.0	0.0190	0.0092	-	-	-
UDMA	2	17	47.1	0.0242	0.0081	0.0171	0.0413	0	2	17	47.1	0.0390	0.0110	0.0293	0.0683	99.9
Organic incubation solution																
BEMA	1	3	100	0.0257	0.0437	-	-	-	1	3	100	0.0468	0.0314	-	-	-
Benzaldehyd	1	1	100	0.5439	-	-	-	-	1	1	100	3.0906	-	-	-	-
BHT	4	14	100	0.0892	0.0147	0.0726	0.1059	93.4	4	14	100	0.1244	0.0190	0.1029	0.1458	96.0
BisEFMA	1	1	100	0.9314	0.1489	-	-	-	1	1	100	1.2615	0.149	-	-	-
BisEMA	3	13	61.5	0.1859	0.0699	0.1068	0.2651	64.0	3	13	69.2	0.2598	0.0619	0.1897	0.3299	48.0
BisGMA	7	38	50.0	0.1431	0.0339	0.1047	0.1814	97.6	6	24	50.0	0.1544	0.0290	0.1216	0.1872	96.8
BL	1	2	100	0.0003	0.0000	-	-	-	1	2	100	0.0006	0.0001	-	-	-
BPA	4	16	68.8	0.1405	0.0980	0.0296	0.2514	77.4	3	11	54.5	0.4660	0.2899	0.1279	0.8041	68.1
BPE	1	1	100	0.4164	-	-	-	-	1	1	100	0.9769	-	-	-	-
CQ	3	18	100	0.1213	0.0120	0.1077	0.1349	40.8	3	18	100	0.1335	0.0117	0.1203	0.1468	94.6
CSA	2	9	100	0.2217	0.0018	0.2197	0.2237	52.3	2	9	100	0.2242	0.0012	0.2228	0.2255	33.3
DCHP	3	4	100	0.0357	0.0481	0*	0.0902	62.3	3	4	100	0.9649	0.2791	0.6490	1.2807	86.3
DDHTP	2	8	100	0.1879	0.0372	0.1457	0.2300	94.4	2	9	100	0.3668	0.0835	0.2724	0.4613	93.5
DEDHTP	1	2	100	0.0000	0.0000	-	-	-	1	2	100	0.0000	0.0000	-	-	-
DEGDMA	1	2	100	0.0516	0.0121	-	-	-	1	2	100	0.2366	0.0101	-	-	-
DMA	1	5	20.0	0.0099	0.0006	-	-	-	1	5	20.0	0.0395	0.0006	-	-	-
DODDMA	1	1	100	0.4410	-	-	-	-	1	1	100	0.6957	-	-	-	-
DMABEE	3	10	100	0.8193	0.2270	0.5624	1.0762	75.2	3	10	100	1.5821	0.3198	1.2202	1.9439	64.6
EGDMA	3	7	100	0.1245	0.0079	0.1156	0.1333	99.3	3	7	100	0.2671	0.0090	0.2569	0.2773	99.6
HDMMA	1	1	100	1.6780	-	-	-	-	1	1	100	2.2338	-	-	-	-
HEMA	3	12	58.3	0.3110	0.0265	0.2809	0.3410	97.7	2	7	28.6	0.8358	0.1051	0.7169	0.9547	99.3
HMBP	3	11	100	2.6034	0.1936	2.3843	2.8225	92.7	3	11	100	4.4073	0.3422	4.0201	4.7945	0
HPMA	1	3	100	6.1439	1.3015	-	-	-	1	3	100	8.1832	1.4730	-	-	-
HQME	1	3	100	0.0001	0.0000	-	-	-	1	3	100	0.0002	0.0000	-	-	-
MAA	1	3	100	0.0045	0.0017	-	-	-	1	3	100	0.1442	0.0192	-	-	-
SDR UDMA	1	1	100	0.3426	-	-	-	-	1	1	100	0.4419	-	-	-	-
TCD-DI-HEA	1	1	100	3.5830	-	-	-	-	1	1	100	6.8688	-	-	-	-
TEGDMA	6	28	60.7	0.1982	0.0155	0.1717	0.2068	96.7	6	29	65.5	0.5425	0.0320	0.5063	0.5787	98.9
TMPTMA	1	1	100	0.3759	-	-	-	-	1	1	100	0.4454	-	-	-	-
TINP	3	7	100	0.2005	0.0647	0.1273	0.2737	0	3	7	100	0.2476	0.0110	0.2351	0.2600	63.3
UDMA	5	26	65.4	0.1761	0.0166	0.1573	0.1948	99.0	5	26	65.4	0.2789	0.0323	0.2424	0.3154	97.9

^*^The lower bound of confidence interval was set as zero, as its actual value was negative while molecule release is a strictly positive parameter

**Table 7:** Weighted means (and SD) for the release measurements collected at 24 h and for the total cumulative release per volume of tested specimens (μmol/mm^3^)

Eluate	24 h release								Total cumulative release							
	Number of studies	Number of records	% > DL	Weighted mean	SD	Lower *CI*	Upper *CI*	*I*^2^	Number of studies	Number of records	% > DL	Weighted mean	SD	Lower	Upper	*I*^2^
Water-based incubation solution																
BHT	4	17	94.1	0.0492	0.0122	0.0354	0.0630	85.3	4	17	94.1	0.1008	0.0133	0.0858	0.1159	88.9
BisEMA	4	24	29.2	0.0337	0.0069	0.0260	0.0415	96.2	4	24	29.2	0.0707	0.0081	0.0615	0.0799	97.8
BisGMA	3	21	9.5	0.0152	0.0066	0.0094	0.0210	75.4	3	21	9.5	0.0298	0.0233	0.0093	0.0502	97.9
BPA	2	8	37.5	0.0420	0.0049	0.0365	0.0475	84.5	2	8	37.5	0.0577	0.0439	0.0190	0.0963	80.8
CQ	4	21	100	0.0594	0.0088	0.0494	0.0694	0	4	21	100	0.0703	0.0097	0.0593	0.0813	68.8
CSA	2	9	100	0.0564	0.0064	0.0492	0.0636	92.0	2	9	100	0.3974	0.0032	0.3938	0.4010	99.2
DEGDMA	1	2	50.0	0.6285	0.1136	-	-	-	1	2	50.0	1.0778	0.1296	-	-	-
DMA	1	5	20.0	0.0123	0.0005	-	-	-	1	5	20.0	0.0537	0.0005	-	-	-
DMABEE	3	10	100	0.1869	0.0519	0.1281	0.2456	29	3	9	100	0.2349	0.0931	0.1296	0.3402	25.7
HDMMA	1	0	0	0.3515	-	-	-	-	1	0	0	1.0545	-	-	-	-
HEMA	2	6	100	0.3697	0.0673	0.2935	0.4459	92.2	2	6	100	0.9145	0.0330	0.8772	0.9518	95.0
HMBP	1	9	100	0.1739	0.0147	-	-	-	1	9	100	0.1906	0.0185	-	-	-
HPMA	1	3	100	1.3600	0.8093	-	-	-	1	3	100	1.4424	0.2187	-	-	-
PI	1	1	100	0.0354	0.0119	-	-	-	1	1	100	0.0544	0.0120	-	-	-
SDR UDMA	1	0	0	0.3515	-	-	-	-	1	0	0	1.0545	-	-	-	-
TCD-DI-HEA	1	1	100	0.2206	0.0196	-	-	-	1	1	100	0.6147	0.0514	-	-	-
TEGDMA	5	26	76.9	0.0314	0.0270	0.0009	0.0619	69.7	5	31	71.0	0.0463	0.0353	0.0063	0.0862	81.8
TMPTMA	1	1	100	0.0689	0.0259	-	-	-	1	1	100	0.1059	0.0163	-	-	-
TPSb	1	6	67.0	0.0297	0.0113	-	-	-	1	6	67.0	0.0525	0.0166	-	-	-
UDMA	2	17	47.1	0.0183	0.0145	0.0055	0.0311	34.7	2	17	47.1	0.0339	0.0064	0.0283	0.0395	81.2
Organic incubation solution																
BEMA	1	3	100	0.0297	0.0511	-	-	-	1	3	100	0.0495	0.0337	-	-	-
Benzaldehyd	1	1	100	0.9351	0.5881	-	-	-	1	1	100	5.3135	0.9264	-	-	-
BHT	4	26	88.5	0.0093	0.0008	0.0083	0.0102	96.5	4	26	88.5	0.0422	0.0091	0.0319	0.0524	98.4
BisEFMA	1	1	100	2.1798	0.3484	-	-	-	1	1	100	2.9525	0.3492	-	-	-
BisEMA	3	13	61.5	0.0763	0.0256	0.0540	0.0987	96.2	3	13	69.2	0.0960	0.0205	0.0782	0.1139	97.8
BisGMA	6	36	47,2	0.1301	0.0266	0.0920	0.1620	97.7	5	20	50.0	0.5376	0.5745	0.0340	1.0412	94.7
BL	1	2	100	0.5385	0.0318	-	-	-	1	2	100	1.1439	0.1329	-	-	-
BPA	4	16	68.8	0.1714	0.1077	0.0495	0.2933	62.4	3	11	54.5	0.6912	0.1324	0.5414	0.8410	21.9
BPE	1	1	100	0.7160	0.0716	-	-	-	1	1	100	1.6796	0.3015	-	-	-
CQ	5	23	100	0.1623	0.0151	0.1452	0.1794	90.8	5	23	100	0.2337	0.0213	0.2096	0.2577	89.4
CSA	3	12	100	0.2858	0.0027	0.2828	0.2888	99.4	3	12	100	0.4022	0.0021	0.3998	0.4046	99.2
DCHP	3	9	100	0.0638	0.0177	0.0437	0.0839	24.7	3	7	100	0.0459	0.0071	0.0379	0.0540	95.1
DDDMA	2	5	100	0.0060	0.0042	0.0013	0.0107	18.7	2	6	100	0.0768	0.0502	0.0200	0.1336	4.2
DDHTP	2	9	100	0.2805	0.0498	0.2241	0.3368	81.3	2	9	100	0.4507	0.0887	0.3504	0.5510	78.1
DEDHTP	1	2	100	0.0432	0.0036	-	-	-	1	2	100	0.0215	0.0033	-	-	-
DEGDMA	1	2	100	0.0077	0.0018	-	-	-	1	2	100	0.0355	0.0015	-	-	-
DMA	1	5	20.0	0.0112	0.0005	-	-	-	1	5	20.0	0.0526	0.0005	-	-	-
DODDMA	1	1	100	0.7581	0.0542	-	-	-	1	1	100	1.1960	0.3597	-	-	-
DMABEE	5	14	100	0.0226	0.0097	0.0116	0.0336	94.1	5	14	100	0.0474	0.0119	0.0340	0.0609	95.9
EGDMA	4	8	100	0.0180	0.0028	0.0148	0.0212	99.7	4	8	100	0.0190	0.0028	0.0159	0.0222	99.9
HDMMA	1	1	100	2.5170	0.2390	-	-	-	1	1	100	3.3508	0.2448	-	-	-
HEMA	3	12	58.3	0.4737	0.0273	0.4428	0.5046	93.4	2	7	28.6	1.1701	0.0668	1.0945	1.2457	98.9
HMBP	4	13	100	0.1143	0.0281	0.0825	0.1462	99.4	4	13	100	0.1471	0.0326	0.1102	0.1840	99.3
HQME	1	3	100	0.1644	0.0170	-	-	-	1	3	100	0.4008	0.0518	-	-	-
HPMA	1	3	100	7.6564	1.6346	-	-	-	1	3	100	10.5940	1.9292	-	-	-
MAA	1	3	100	0.0082	0.0030	-	-	-	1	3	100	0.2617	0.0348	-	-	-
SDR UDMA	1	0	0	0.3515	-	-	-	-	1	1	100	1.0141	0.1110	-	-	-
TCD-DI-HEA	1	1	100	6.5590	0.4471	-	-	-	1	1	100	12.5738	0.5209	-	-	-
TEGDMA	5	26	61.5	0.2265	0.0204	0. 2035	0.2140	99.1	6	29	65.5	0.7651	0.0433	0.7161	0.8141	99.2
TINP	5	15	73.3	0.1755	0.0350	0.1359	0.2151	54.8	5	15	73	0.4215	0.0185	0.4006	0.4424	70.5
TMPTMA	1	1	100	0.6463	0.2068	-	-	-	1	1	100	0.7658	0.1385	-	-	-
TPP	1	1	100	0.0006	0.0006	-	-	-	1	1	100	0.0018	0.0004	-	-	-
TPSb	2	7	71.4	0.0020	0.0017	0.0001	0.0039	49.6	2	7	71.4	0.0347	0.0167	0.0158	0.0536	92.1
UDMA	5	26	65.4	0.1886	0. 0193	0. 1668	0.2496	98.0	5	26	65.4	0.2976	0.0373	0.2554	0.3397	98.3
2-PEA	1	1	100	0.0138	0.0071	-	-	-	1	1	100	0.0755	0.0193	-	-	-

In such cases (BPA, DEGDMA, HPMA), where a high value of SD could be observed, a great heterogeneity of the original data could be assumed. For many eluates, a considerable heterogeneity of the resulting data (*I*^2^ > 75%) was evident, which might be due to several factors, such as different types of materials, materials manufacturers, study protocols, and analytical methods used to determine the amount of substance eluted together with various limits of detection that often had to be calculated.

**Fig. 2:**
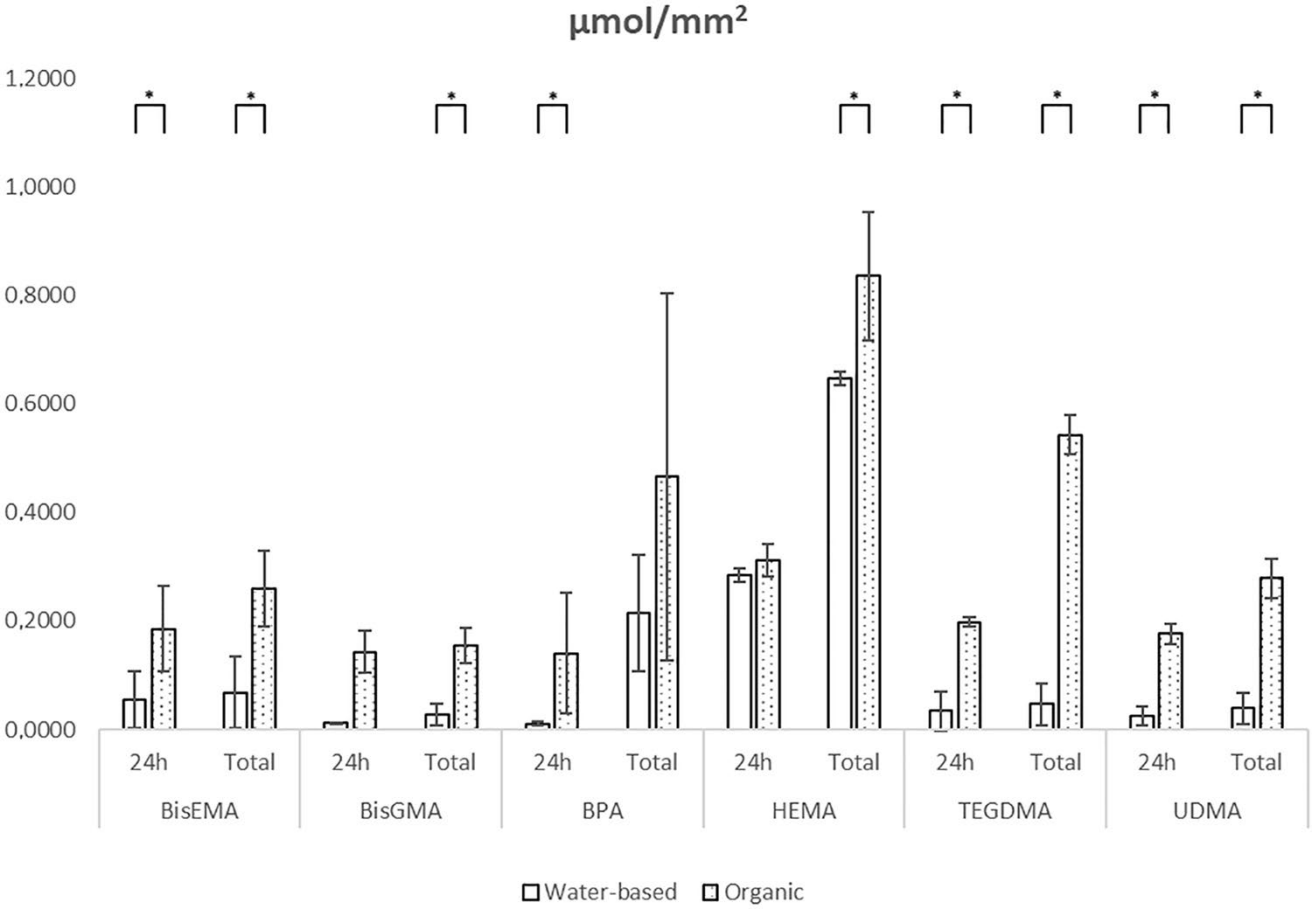
Elution per sample surface (µmol/mm^2^) of the most detected monomers in organic and water-based solvents

**Fig. 3:**
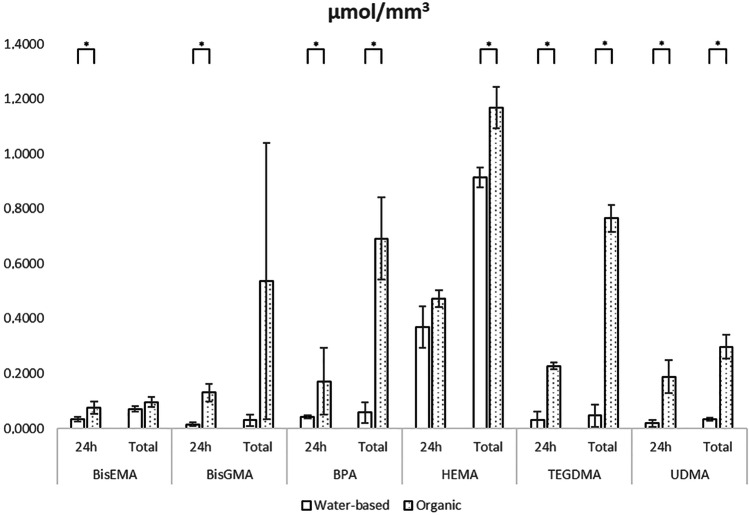
Elution per sample volume (µmol/mm^3^) of the most detected monomers in organic and water-based solvents

The results were divided into two groups of water-based (distilled water, artificial saliva, and different types of cell media) and organic (methanol, ethanol solution) incubation solvents. In the majority of the results, a statistically significant difference between those two groups was detected, with a superior elution in organic solvents for most monomers. Bis-EMA, Bis-GMA, BPA, HEMA, TEGDMA, and UDMA were the most detected monomers, and their elution in organic and water-based solvents is summarized in Figs. 2 and 3. Conversely, for most additives, the results were rarely reported in more than one study. In some cases, the value was even derived from only one sample in a single study; therefore, the values of SD and heterogeneity could not be calculated for such molecules. Among the monomers, HEMA demonstrated the highest elution in both types of solvent (both per surface and per volume). A slightly reduced elution was recorded for TEGDMA, BPA, and Bis-EMA, depending on the solvent nature. Bis-GMA showed the lowest release (per volume and per surface) irrespective of the solvent, and a very low release was detected also for UDMA. The release of some additives appeared similarly high. For example, DMABEE (ethyl 4-dimethylamino benzoate), CQ (Camphoroquinone), CSA (Campheracid anhydride), BHT (Butylated hydroxytoluene), HMBP (2-hydroxy-4-methoxybenzophenone), and TINP (Tinuvin P) showed to elute at levels comparable to those of some monomers, especially in organic solvents.

The Pearson’s correlation test demonstrated a strong significant inverse correlation between the amount of released molecule and the molecular mass in organic solvents per surface (*r* =  − 0.887; *p* = 0.046) and very strong correlation per volume (*r* =  − 0.914; *p* = 0.011). Such correlation was not significant in water-based solvents (*p* > 0.05).

For results expressed as monomer elution per surface (μmol/mm^2^), a weak positive correlation was detected between the total surface area of the specimen and the amount of released eluate, both when data coming from all the different types of solvents were pooled together (*r* =  − 0.283; *p* = 0.025) and for organic solvents alone (*r* =  − 0.303; *p* = 0.037); such correlation was not evident when just water-based solvents were concerned (*p* > 0.05). For results expressed as monomer elution per volume (μmol/mm^3^), the same weak positive correlation between the release amount and the specimen surface was found when data coming from both types of solvents were pooled together (*r* = 0.0.223; *p* = 0.027). Such a correlation was detected even when the organic solvents were considered separately (*r* =  − 0.302; *p* = 0.022), but it could not be confirmed (*p* > 0.05) for the results independently coming from the water-based solvents.

A weak positive correlation was found between the sample volume and the amount of released eluate per volume (μmol/mm3) considering data from organic solvents (*r* =  − 0.278; *p* = 0.036), while there was not any correlation when considering water-based solvents (*p* > 0.05).No correlation (*p* > 0.05) was detected between the release of monomers (both per surface and per volume) and the volume of solvent, independently on the solvent nature.

## Discussion

The present meta-analytical review included in vitro studies with the following common research protocol: preparation of resin-based dental materials specimens, soaking into different types of water-based or organic solvents (without any pre-incubation treatment), and quantification of the released compounds at different time intervals, with reference analysis at 24 h.

To determine the quantity of released compounds, the included studies performed the analysis prevalently through the HPLC (high-performance liquid chromatography) or GC–MS (gas chromatography mass spectrometry) methods. The analytical methods of LC–MS (liquid chromatography-mass spectrometry) and UPLC-MS/MS (ultraperformance liquid chromatography-tandem mass spectrometry) were used rarely. All the above-mentioned chemometric techniques are advanced methods able to detect singular molecules if used with correct calibration. HPLC and LC–MS are very versatile and popular methods because of their wide range of applications and their ability to detect molecules with high molecular weight [107]. Both UPLC- MS/MS and GC–MS (performed strictly with vaporized, volatile, and thermally stable molecules) [108] are instead particularly suitable methods for low molecular weight particle analysis [107].

The elution of a total of 36 substances could be observed within the included studies. The majority of data referred to monomers. Monomers are a significant component of resin-based composites as they represent about 20–40% of their content. Undesirable effects are attributed to monomers, which are released during incomplete monomer-polymer conversion [109]. The inorganic fillers as quartz, borosilicate, lithium–aluminum–silicate glasses, and amorphous silica, represent about 60–80% of resin-based composite content, but they do not seem to play a major role in the biocompatibility of these materials [5]. Additives, that usually play a role in promotion, modification, or inhibition of the polymerization reaction, represent only about 1–3% of the composition. Manufacturers are not obliged to disclose the ingredients in the composition of the materials if they do not exceed 1% of total volume [89]. Besides that, Material Safety Data Sheets (MSDS) of products are often incomplete [110, 111].

In the present study, HEMA (2-hydroxyethyl methacrylate) was the most released monomer. It can be described as a low molecular mass monomer (130 g/mol) with small dimensions, highly soluble in both types of solvents. Actually, in case of HEMA, no statistically significant difference between the release in organic and water-based solvents at 24 h was observed (Figs. 2 and 3). Due to its hydrophilic character, HEMA is a co-monomer frequently added in commercial resin-based materials in order to prevent the separation between water and hydrophobic co-monomers [112, 113]. On the other hand, some negative physic-mechanical features of HEMA (as low degree of conversion and water retention impairing a good polymerization) were reported [114]. Moreover, HEMA demonstrated a certain degree of cytotoxicity affecting cell viability [115–117], which might be aggravated by HEMA water solubility. Hydroxyethyl acrylamide (HEAA) and diethyl acrylamide (DEAA) are regarded as the two most promising alternatives to HEMA [114].

TEGDMA, BPA, and Bis-EMA revealed a quite high solubility (depending on the solvent) as well. TEGDMA demonstrated relatively high levels of release, specifically in organic solvents. TEGDMA is a low viscosity and low molecular mass (286.32 g/mol) molecule often added into composite materials in order to reduce the viscosity of the mixture [118, 119] and thus increase the degree of conversion (DC). Unfortunately, the higher DC determined by TEGDMA also increases the polymerization shrinkage of the material [120]. For this reason, TEGDMA is often at least partially substituted by another monomer of higher molecular mass and lower viscosity (for example Bis-EMA) [119]. Cytotoxic effects of TEGDMA on gingival and human fibroblasts clinically related to pulp inflammation and necrosis were reported [121, 122].

BPA demonstrated quite high values of release in results expressed as substance elution per volume (μmol/mm^3^), and significantly higher release was detected in organic solvents. Despite BPA is not directly present in resin-based composites, it still occurs in form of impurities [123]. Bis-DMA (bisphenol A dimethacrylate) can be converted into BPA by hydrolysis after its exposure to the esterase enzymes contained in saliva [124, 125]. Although Bis-GMA (synthetized from BPA and glycidyl methacrylate) does not undergo this reaction presumably because of its chemical structure which prevents hydrolysis at the ester linkage [118], Bis-GMA-based materials still showed detectable BPA release [126–128]. BPA molecule is insoluble in water but demonstrates a good solubility in organic solvents as alcohols, ethers, and fats [129]. BPA molecular mass corresponds to 228.28 g/mol. The European Food Safety Authority (EFSA) stated in 2015 that the temporary Tolerable Daily Intake (TDI) for BPA is 4 μg/kg/day [130]. During the measurements of a human BPA exposure, it was estimated that an average human organism directly or indirectly receives about 30.76 ng/kg per body weight per day [131–134]. Resin-based dental restorations are listed among the main sources of oral BPA intake [123, 135–137]. The negative human health effects of BPA are related to its endocrine disrupting activity [138–142] and have greater impact in early-life exposure [140, 143]. Alternative forms of bisphenol, as BPS (bisphenol S) and BPF (bisphenol F) were introduced to substitute BPA in order to avoid such an endocrine-disrupting chemical, but recent studies referred a similarly unfavorable behavior [142, 144].

High values in results expressed as monomer elution per surface (μmol/mm^2^) were detected for Bis-EMA in both types of solvents but the release was significantly increased in organic solvents. Bis-EMA is a hydrophobic analog of Bis-GMA used as a basis monomer of several dental resin materials in order to reduce their viscosity [17]. Low viscosity is caused by the absence of free hydroxyl groups which allows major incorporation of inorganic filler [47]. Bis-EMA is a low volatile hydrophobic molecule with a molecular mass of 452 g/mol. It was reported that also Bis-EMA containing materials released BPA as an impurity resulting from Bis-EMA degradation [128].

The records for UDMA release were generally very low, especially in water-based solvents. UDMA is another co-monomer commonly applied in dental resin-based materials to enhance the viscosity. Considered to be an alternative to Bis-GMA [145], UDMA usage is limited by its high molecular mass (470.56 g/mol), which results in a remarkable volumetric shrinkage clinically related to greater marginal gap between tooth and restoration [120]. Regarding its cytotoxicity, it has been reported that UDMA inhibits cell growth in vitro at the concentration of 0.1 mM [146].

The lowest release was demonstrated for Bis-GMA, which might be explained by its high molecular mass (512.599 g/mol) and very slight solubility in all types of solvents. Bis-GMA is a BPA derivate that is most frequently used as the base of resin-based composites. Bis-GMA molecule is composed by methyl methacrylate groups added to the hydroxyl groups of BPA via a glycidyl spacer [147]. Bis-GMA is a base matrix compound generally convenient for its low volumetric shrinkage after polymerization, good mechanical properties, high refractive index, low volatility, and diffusivity into tissues and excellent adhesion to enamel [127, 148]. Great voluminosity, strong molecular interactions driven by H-bonding, and large molecular mass are the determinants of its particularly high viscosity [149]. Nevertheless, the raised doubts about Bis-GMA low viscosity that might negatively affect mechanical properties of materials [150] and its possible cytotoxic effects linked to BPA [151, 152] have started the search for alternatives and led to the marketing of Bis-GMA resin-based materials [147, 153], such as Bis-EFMA-based composites [99].

The sequence of release of the substances corresponds to the order of their cytotoxic potential assessed by Reichl et al. [154]: HEMA < TEGDMA < UDMA < Bis-GMA. In that study, a 50% reduction in cell viability was reported after exposure of human gingival fibroblasts to Bis-GMA at a concentration of 0.087 mmol/L, to UDMA at 0.106 mmol/L, and for HEMA at 11.530 mmol/L. For TEGDMA, such viability decrease was detected at 3.460 mmol/L. The reduction of cell viability was related to the increased amount of reactive oxygen species and oxygen stress, and to DNA strand damage and cell cycle alterations [109]. Comparing the dental resin-based materials containing or non-containing Bis-GMA, a greater cytotoxic and genotoxic potential of materials releasing Bis-GMA and TEGDMA was observed [122]. The concrete effects of monomers applied in direct contact with dental pulp cells, as inflammation and inhibition of dentin mineralization, were described in many studies [155–157].

Among the included studies, the release of many additives was detected as well. Although the additives are present in the composition of resin-based materials only in a small percentage, some still showed quite high release, reaching the levels of frequently eluted monomers (Tables 6 and 7). However, the results for additives need to be considered with caution, as only few studies analyzed their elution and therefore the input data were not as strong as for monomers.

In the present study, results were given both for the elution at 24 h and for total cumulative release. The analysis at 24 h was considered the reference, as it is quite common in many studies and was present in all included studies. Concerning the total cumulative release, it must be underlined that the cumulative time period varied among the different studies from days to months.

The release among the included studies was confronted in different solvents. Most of the studies tested the materials in more than one solvent, prevalently diluted ethanol, distilled water, and methanol. Some protocols also tested elution in artificial saliva and in various types of media commonly used for cell culture growth. Most but not all studies clearly specified if the solution was refreshed after every analysis or if the results were of cumulative character. Water-based solvents as artificial saliva or distilled water can mimic intraoral conditions. Organic solvents are characterized by a major dissolution efficiency probably ascribable to their better penetration, sorption, and swelling of the polymer material [18]. Based on the outcome of this study, there was a significant difference between elution of water-based and organic solutions (Figs. 2 and 3), which offered a prevalently better environment for greater elution. Since monomers are generally hydrophobic, and the original studies declared similar differences between the major release in organic solvents and water-based ones, this outcome only confirmed the expectations.

Examining the influence of molecular mass on the amount of released molecules, a strong negative correlation was reported. This would mean that small molecular mass molecules tend to have greater mobility and polarity, and to release faster and more easily, unlike heavier and larger molecules. Such supposition corresponds to the results of the current paper considering the release of the six mainly examined monomers. HEMA, as a very light molecule, was released the most, followed by TEGDMA, BPA, and Bis-EMA. Lower release was detected for UDMA, and even lesser for Bis-GMA, the molecule with the highest molecular weight among these monomers.

The importance of the surface area exposed to the solvent was confirmed by the weak but statistically significant correlation between the amount of released molecule and the surface of specimens in the results for both surface and volume; this was valid for the results of both the type of solvents pooled together, and also for the organic ones separately. A faster elution from surface and subsurface layers compared with deeper layers was reported in previous studies [43, 158]. Therefore, it can be hypothesized that the more extensive the surface of the restoration is, the higher the risk of monomer elution becomes. Such theory might have an interesting clinical impact. Considering the minimal amount of intraorally polymerized resin-based material used for the cementation of indirect restorations, the created surface of material exposed to oral environment is minimal. From this point of view, indirect restorations would be the optimal solution regarding to the risk of monomer release and potential toxicity. Only two studies [92, 100] specified the actual surface area that was in contact with the solvent without considering the area contacting the bottom of the container all the time during the soaking phase. The rest of the studies did not mention this aspect.

A weak correlation between the release and the volume of specimens was detected when data for both solvents were pooled together. Such correlation may sustain the hypothesis that the major volume and so the major thickness of the material increment determines the major release. The descending efficiency of polymerization with the increasing thickness increment of cured material led to incomplete polymerization and reduced the degree of conversion in the deep layers. This results in the persistence of free monomers and their potential subsequent release [159, 160].

A correlation between the release of substances and the absence or presence of oxygen inhibition layer (OIL) could not be determined, as there were not enough data for such calculation. Only few studies [53, 90, 91, 99, 100] provided results in the presence of OIL; all the remaining studies clearly specified the steps taken to prevent the presence of the OIL (blocking the contact of the surface of material with oxygen before the curing process through glass, matrix strip, or glycerin gel). The presence of atmospheric oxygen may inhibit the correct polymerization of monomers and create a surface layer of unreacted monomers. The decrease of DC in the presence of the OIL was demonstrated [161], as well as an increase of DC after removing the OIL by polishing and finishing [162]. The correlation seemed to confirm the hypothesis that the release of monomers would be higher in the presence of OIL.

In general, the results of the current review are broadly consistent with Van Landuyt et al. [18] 10-year-old findings. However, slight differences deserve to be mentioned. The present work showed a statistically significant positive correlation (although weak) between the amount of released molecule and the specimen volume, which was not evident in Van Landuyt et al. article. In turn, their review revealed a weak but significant (positive) correlation between the released amount of each eluate and the amount of solution in which the resin-based specimen was immersed, which our findings failed to demonstrate.

The present meta-analytical review provided an exhaustive summary of the current evidence regarding substances elution from resin-based dental materials in vitro. Therefore, taking into account all the inherent drawbacks of in vitro studies, caution is recommended in generalizing the above-mentioned results. In vitro experiments regarding dental materials and their properties usually tend to mimic the environment of oral cavity, maintaining the reproducibility and stability of applied analytical methods. In the oral cavity, there is a constant influx of new saliva, which washes the surfaces of tooth and restorations, and which is subsequently drained by swallowing. The composition of natural human saliva is very complex and variable, depending on several individual factors (such as food intake, bacterial colonization, and others), which fundamentally affect intraoral pH. For those reasons, it is not exactly possible to create a synthetic formula identical to natural saliva [163]. However, the use of natural human saliva is unreliable as well, due to its lack of stability outside the oral cavity [164]. In case of monomer elution research, it would be therefore necessary to ensure an extraction solvent that resembles natural saliva and its constant exchange. It could be speculated that refreshing the extraction solvent could prevent reaching the chemical balance that progressively slows down monomer elution, thus letting this phenomenon run naturally without restrictions. On these bases, the protocols where the solvent was periodically refreshed after each measurement adhered more closely to the real intraoral conditions [18]. Nevertheless, reproducing the precise intraoral conditions seems very difficult, and this should be taken into account when evaluating the results of in vitro studies that may not fully correspond to the in vivo situation.

Finally, besides all the inherent drawbacks of in vitro studies, other potential limitations to the present review were related to the lack of standardization of the included studies: variability of original units of measurement, specimens size, samples polymerization and storage, and absence of limit of detection values, in fact, complicated the paper comparison.

The variability of the original units of measurement and their conversion into a common one were among the most important issues that needed to be solved in order to obtain uniform outcomes which could be subsequently compared and analyzed. The primary results were expressed mostly in concentration, while some of them were already related to surface and volume. The choice to express the outcomes into common units, moles per surface and volume, was based on the considerations given by Van Landuyt et al. [18]. These authors preferred this approach in order to express the amount of released molecules per surface and volume that may be confronted with actual dimensions of in vivo restorations.

There were other points in the selected original studies which were rarely standardized, thus making the comparability of the studies more difficult. Although the specimens were mostly disk-shaped, their size varied in every study. The details about the setting of the curing unit, the polymerization time (respecting or not the time recommended by the manufacturer), and the distance of the curing unit from the specimen surface were not always complete. Only some studies [62, 83, 84, 92, 94, 96–98, 101, 102, 165] provided very detailed information about the storage conditions during the soaking phase, namely the exact temperature and light-conditions. However, a clear specification of the solvent characteristics and its volume was one of the most important information that was required in the present review, and, if not clearly specified, represented an exclusion criterium. In the included papers, the specimens were soaked into the extraction liquid almost immediately, without any pre-incubation period.

Another important element that was often missing among studies was the limit of detection value. It was decided to supplement the original results under the limit of detection by the value of limit of detection itself. In case this was missing, then the lowest measured result within the included studies for the given molecule was used as a substitute value. This approach permitted not to lose but valorize data of measurements under the limit of detection; however, it might also represent a minor source of inaccuracy for the primary results. Moreover, some authors provided the results of their studies only in the form of graph and not in exact numbers. Only the studies where those graphs were clearly legible were included. Although a precise digital graphical tool was used to extract numerical values, this procedure might have still led to a minimal additional inaccuracy in obtained data.

In light of the above-mentioned limitations, the authors of the present paper emphasize the necessity for standardization, as already underlined by Van Landuyt et al. Despite their 10-year-old attempt to suggest several guidelines for study design standardization, in fact, no evident improvement has been detected in the literature regarding monomer elution from resin-based dental materials of the last 10 years. The authors of the present study recommend, therefore, that future works follow the above-mentioned guidelines and reaffirm the need to overcome the great heterogeneity ruling in the literature through the standardization of the following points: dimension and form of the specimen, specimen manufacturing protocol (including polymerization conditions such as intensity, modality, time of polymerization, and prevention of OIL), volume and types of soaking solvent (pH), soaking conditions (temperature, light, and real specimen surface exposure to the solvent), methodology of analysis, and units of measurement to quantify the results.

## Conclusions

Based on the findings of this meta-analytical systematic review, it was possible to draw the following conclusions:

1. The difference of monomer elution between organic and water-based solvents was evident, as organic solvents provoked major substances release.
2. The strength of molecule elution is negatively correlated to its molecular mass: a lighter molecule elutes more, while molecule with higher molecular mass is released less.
3. The surface area of specimen plays an important role: the major release of substances was observed in specimens of more extended surface.
4. The volume of specimen may be an important factor as well: the more voluminous specimen demonstrated slightly major elution tendency.

It must be underlined, however, that the comparison of the different studies was not always effortless, given the wide variability of protocol set-ups, methodologies, and materials. In order to improve the homogeneity of future studies, it would be advisable to standardize the following aspects for in vitro experimental protocols on composite elution: specimen size, polymerization setting, solvent character, analytical method and results expressed numerically.

## Supplementary information

Below is the link to the electronic supplementary material.

Supplementary file1 (XLSX 15 KB)
